# Reply to Mortazavi, “Acquired Antibiotic Resistance in *Escherichia coli* Exposed to Simulated Microgravity: Possible Role of Other Space Stressors and Adaptive Responses”

**DOI:** 10.1128/mBio.00391-19

**Published:** 2019-03-26

**Authors:** Madhan R. Tirumalai, George E. Fox

**Affiliations:** a Department of Biology and Biochemistry, University of Houston, Houston, Texas, USA

**Keywords:** *Escherichia coli*, antibiotic resistance, microgravity

## REPLY

We thank Dr. Mortazavi for pointing out the role of radiation as an important factor under microgravity conditions (1) while referring to our paper (2). We agree with Dr. Mortazavi that radiation is an important component when it comes to assessment of bacterial response to the space environment. We have previously published papers on radiation-resistant spore-producing *Bacillus* strains isolated from spacecraft cleanroom facilities that are of planetary protection concern (3–7). In doing this, we have observed that the bacterial adaptive response can be of two kinds: changes in gene expression in response to the environment and changes which are genomic. Overall, we agree that studies on microbial adaptation examining the long-term effects of simulated microgravity in combination with radiation would be significant. If done using simulated microgravity, such studies would avoid the significant cost of performing similar studies on bacteria in space (14).

In fact, we have already attempted to do this with the Escherichia coli MG1655 strain exposed to both simulated microgravity and radiation. To accomplish this, a model radiation environment was produced using radioactive cobalt wires (Co-60) suspended equidistant from the center of an incubator emitting gamma rays. This work was done in collaboration with Dr. John Ford and then-student Emma Howard Schulze at the Department of Nuclear Engineering at Texas A & M University in College Station. Unfortunately, we could not complete the study due to logistical constraints that required the experiment to be terminated after only 200 generations.

Resequencing the genome of the E. coli MG1655 strain exposed to 200 generations of both microgravity and radiation resulted in the identification of only two mutations in known genes that could be related to the radiation exposure. The first gene, *recD*, promotes homologous recombination in the repair of double-strand DNA breaks and during bacterial conjugation, as part of the alternative end-joining (A-EJ) system (8, 9). The second gene, *mrdB*, encodes an inner membrane protein that is involved in the synthesis of a cylindrical peptidoglycan which plays a role in cell shape, elongation, and division (10, 11). Both these genes are also implicated in antibiotic resistance (12, 13). However, both the mutations resulted in changes in the domains of the respective protein products that do not affect their functions. Given the limited scope of the study, the results obtained are insufficient to derive a comprehensive picture.

We once again thank Dr. Mortazavi for his comments. We do recognize that such studies should be extended toward further long-term exposure to both radiation and simulated microgravity and that doing so is essential to obtain a more holistic systems-level picture of microbial adaptation to space conditions.
